# Unilateral Thyroid Hypoplasia With Isthmic Absence and Presence of Pyramidal Lobe and Levator Glandulae Thyroidae: A Cadaveric Case Report

**DOI:** 10.7759/cureus.112244

**Published:** 2026-07-07

**Authors:** Kavita Singh, Rajani Singh, Rabiya Bashri

**Affiliations:** 1 Physiology, Graphic Era institute of Medical Sciences, Dehradun, IND; 2 Anatomy, Graphic Era Institute of Medical Sciences, Dehradun, IND

**Keywords:** agenesis of isthmus, hypoplasia of the thyroid, levator glandulae thyroideae, pyramidal lobe, thyroid gland

## Abstract

The thyroid gland is a very essential endocrine gland as it regulates the growth and development of individuals, along with controlling metabolism. However, oftentimes, it is subjected to congenital anomalies, like agenesis of its isthmus, hypoplasia, presence of the pyramidal lobe, and a fibro-muscular strip called levator glandulae. These anomalies may culminate in various complications during and after thyroid surgeries. This underscores the importance of these anomalies for thyroid surgeries. The aim of the study is to describe the congenital anomalies, i.e., hypoplasia, agenesis of isthmus, presence of the pyramidal lobe, and levator glandulae thyroidae. During a routine dissection of the head and neck for first-year MBBS students, the authors detected hypoplasia, agenesis of isthmus, presence of the pyramidal lobe, and levator glandulae thyroidae in a male cadaver of 60 years. The occurrence of these anomalies in the same individual is a rare finding. The hypoplasia of one lobe usually is asymptomatic but sometimes may be linked to hypothyroidism. Similarly, individuals with absent isthmus are euthyroid but may be associated with hypo- or hyperthyroidism. Similarly, the pyramidal lobe, if left behind, may be affected by the same diseases again, for which thyroidectomy was performed. Hence, detailed knowledge of these congenital anomalies is of immense use to thyroid surgeons to minimize intra- and postoperative complications.

## Introduction

The thyroid gland, a very crucial endocrine gland, is involved in the growth and development of an individual. Conventionally, the thyroid gland comprises two lobes connected by the isthmus [1]. However, various congenital anomalies of the thyroid gland, like the lingual thyroid, sublingual thyroid, ectopic thyroid tissue, absence of one lobe, absence of the isthmus, and hypoplasia, are described in the literature [2]. The absence or underdevelopment of one of the lobes or isthmus may cause impairment of the functions of the thyroid, depending upon the degree of the deficit of thyroid tissue [2]. Hypoplasia of the thyroid gland refers to the underdevelopment of the lobes of the thyroid gland unilaterally or bilaterally, which may or may not be in functional condition [2]. However, the prevalence of hypoplasia involving the thyroid gland is extremely low, amounting to approximately 0.02-0.05% in the general population [3]. There is a paucity of literature on this anomaly of the thyroid gland, especially from South Asian countries [3-4]. In addition, the absence of the isthmus, which connects the two lobes of the thyroid, is another anomaly related to the thyroid gland with a prevalence of 0.05-0.2% [5]. As is imminent from the above description, both these anomalies of the thyroid gland are very rare entities.

Sometimes, an additional lobe called the pyramidal lobe may also be present. Its incidence varies between 18% and 66% [6]. However, a recent meta-analysis reported a 42.82% prevalence with predominance in males as compared to females [7].

Moreover, occasionally, a fibromuscular strip called the levator glandulae thyroidae connecting the hyoid bone and isthmus or pyramidal lobe is documented in the literature [8-9], with an incidence of 5-19.8% [9]. 

However, in the present cadaver, we observed unilateral hypoplasia of the right lobe, along with the absence of the isthmus, a pyramidal lobe arising from the left lobe, and levator glandulae thyroidae connecting the pyramidal lobe with the hyoid bone, co-occurring in a male cadaver aged 60 years, which again is an exceptionally rare finding. The occurrence of all the abovementioned anomalies in a single thyroid gland is extremely rare.

This case report highlights the abovementioned anomalies occurring together in the same cadaver along with the clinical significance of the same.

## Case presentation

During routine dissection of the head and neck for teaching MBBS students of the Phase I 2025-2026 batch, the authors observed multiple variations in the thyroid gland of a formalin-fixed 60-year-old male cadaver. The neck was dissected by the conventional dissection method elaborated in Cunningham’s dissecting manual. After reflecting the infrahyoid muscles, the thyroid gland was exposed.

**Figure 1:**
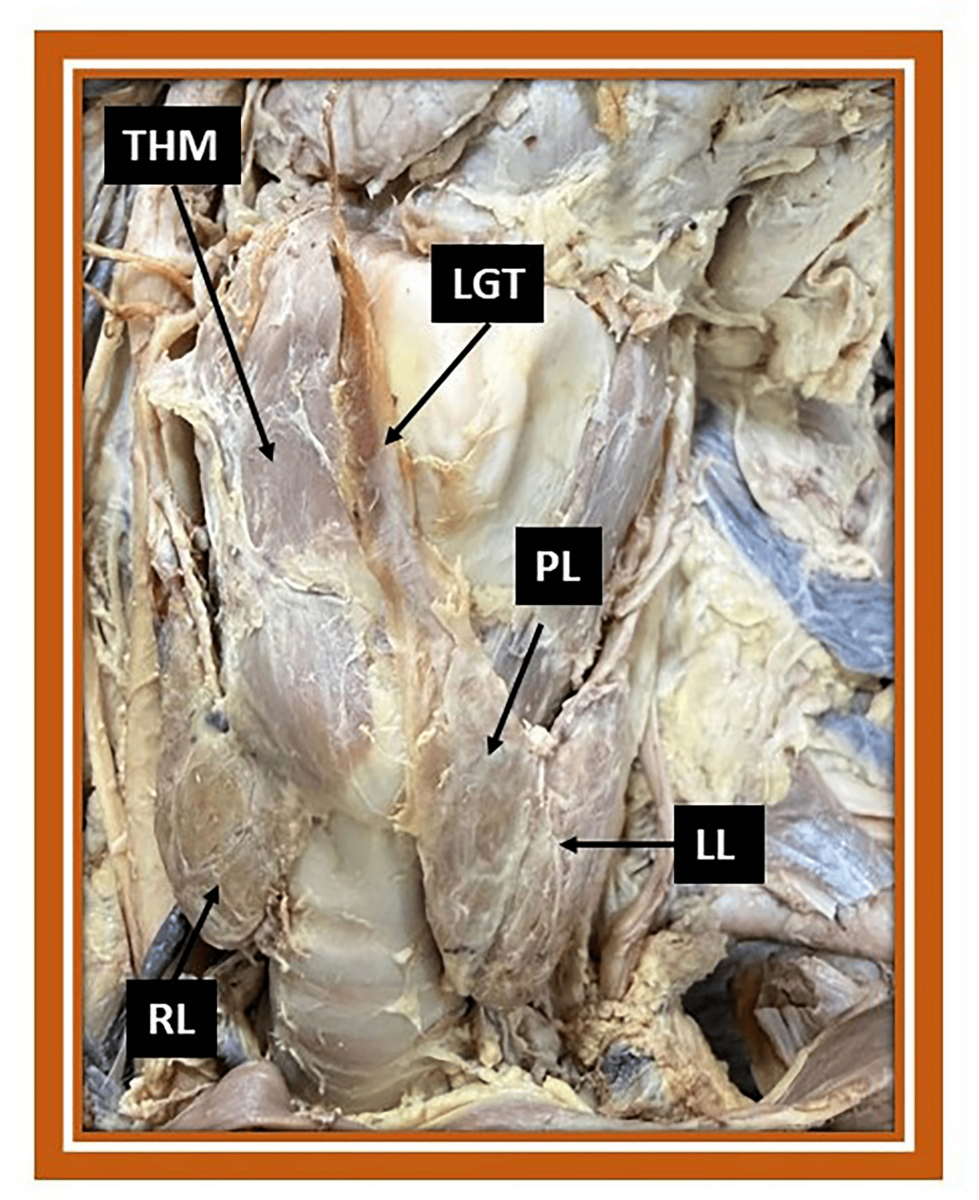
Photograph demonstrating the right thyroid lobe hypoplasia, absent isthmus, pyramidal lobe arising from the left lobe, and levator glandulae thyroidae extending to the hyoid bone. THM: thyro-hyoid muscle; LGT: levator thyroidae glanduae; PL: pyramidal lobe; LL: left lobe of the thyroid; RL: right lobe of the thyroid

The following variations of this thyroid gland were observed:

First, on visual observation, the right lobe was smaller than the left lobe. On measuring the dimensions, the maximum length of the right lobe was 4.0 cm, while that of the left lobe was 4.6 cm. The maximum width of the right lobe was 2.0 cm, and that of the left lobe was 3.0 cm, indicating hypoplasia of the right lobe of the thyroid gland (Figure 1). The thickness of the right lobe was 1.8 cm, and that of the left lobe was 2.5 cm

Second, the isthmus connecting the two lobes was absent. Then, the third lobe, the pyramidal lobe originating from the left lobe, was noted. Fourth, the fibro-muscular strip extending from the tip of the pyramidal lobe to the hyoid bone, measuring 6.0 cm, was also detected.

All these variations of the thyroid gland were detected in the same cadaver.

## Discussion

In standard conditions, the thyroid gland comprises two lobes, namely, the right and left lobes, and these two lobes are connected by horizontal thyroid tissue known as the isthmus. However, some studies have reported absence of the isthmus [5]. In the present case also, the isthmus connecting the two lobes of the thyroid gland was found to be absent. The literature suggests that agenesis of the isthmus may be associated with mutations in genes, namely, TITF1, PAX8, and FOXE1/TITF2 [10].

Embryologically, the distal end of the thyroglossal duct obliterates, but when it persists, it forms a pyramidal lobe. 

In addition to the normal two lobes, occasionally, a third lobe called a pyramidal lobe may also be present, which may arise from either the right lobe, left lobe, or isthmus. The pyramidal lobe is not a constant finding but was observed in 18-66% of individuals [6]. This extra lobe may arise from either the right and left lobe or the isthmus. In our case, this lobe arose from the left lobe of the thyroid gland. As per one recent study, this additional lobe is found predominantly in males [7]. In the current report, the pyramidal lobe was also observed in a male cadaver.

Another developmental variation associated with the thyroid gland is the presence of a fibromuscular band known as the levator glandulae thyroidae. This structure connects the hyoid bone either with the isthmus or pyramidal lobe [8]. In the present case, this fibromuscular strip extended between the hyoid bone and pyramidal lobe. Comparing the dimensions of the two lobes of the thyroid gland revealed hypoplasia of the right lobe in the present case.

The hypoplasia of one of the lobes and absence of isthmus are extremely rare variations, while the rest of the variations, i.e., presence of the pyramidal lobe and levator glandulae thyroidae, are not so uncommon, but all these anomalies co-occurring in the same individual is a really uncommon finding.

Correlation of the variations of the thyroid gland under study with clinical implications

The agenesis of the isthmus of the thyroid gland as such is asymptomatic but may be associated with autoimmune thyroid nodules, thyroiditis, and primary thyroid cancer [10]. If the absence of the isthmus is detected, thyroid nodules and thyroiditis should be excluded. Moreover, the existence of the pyramidal lobe or levator glandulae thyroideae should be ruled out during complete thyroidectomy [10].

The thyroid gland develops from the two divisions of the thyroglossal duct. It is postulated that agenesis of the isthmus occurs when this division occurs at the level cranial to normal bifurcation [10]. Individuals with absent isthmus are reported to be euthyroid, but in certain people, this condition is associated with hypothyroidism or hyperthyroidism [11].

The presence of the levator glandulae thyroideae is important for surgeons during cricothyroidotomy, percutaneous tracheotomy, thyroidectomy, and drainage of thyroglossal duct cysts, as it may be inadvertently injured during these interventions [12].

The pyramidal lobe is reported to occur in 15-75% of the population [13]. The pyramidal lobe contains normal functional thyroid tissue. The importance of this lobe lies in the fact that it may press structures, such as the laryngeal nerve. Moreover, it may be accompanied by other congenital anomalies of the thyroid gland [14], which should be ruled out.

## Conclusions

The thyroid gland is an important gland of the human body that regulates metabolic activities, as well as somatic and psychic growth. However, it is very often subjected to various types of congenital anomalies. Out of these congenital anomalies, the agenesis of the isthmus, hypoplasia, presence of a pyramidal lobe, and levator glandulae thyroidae are some important congenital anomalies of the thyroid gland with varying incidences. Hypoplasia and absent isthmus are extremely rare anomalies, although the other two anomalies, i.e., presence of the pyramidal lobe and levator glandulae thyroidae, are not so rare. These anomalies are mostly described to occur separately, but, in our study, these were observed in the same specimen, which is again very rare. These anomalies should be kept in mind while performing thyroid surgeries and managing thyroid disorders to minimize the risk of mismanagement and decrease the rate of mortality and morbidity. The knowledge of the anomalies described in the present case may help radiologists in avoiding misinterpretation of radiographs.

## References

[1] Watkinson J, Gleeson M (2021). Gray’s Anatomy: The Anatomical Basis of Clinical Practice.

[2] Butt N, Ausama Khan A, Mirza DN (2025). Unilateral thyroid hypoplasia in a euthyroid adult: a rare congenital anomaly. Cureus.

[3] De Sanctis V, Soliman AT, Di Maio S (2016). Thyroid hemiagenesis from childhood to adulthood: review of literature and personal experience. Pediatr Endocrinol Rev.

[4] Szczepanek-Parulska E, Zybek-Kocik A, Wartofsky L, Ruchala M (2017). Thyroid hemiagenesis: incidence, clinical significance and genetic background. J Clin Endocrinol Metab.

[5] Hamiedah MM, Al-Bdour B, Alshoubaki WI (2024). Thyroid isthmus agenesis coinciding with papillary thyroid carcinoma: a case report. Int J Adv Med.

[6] Mortensen C, Lockyer H, Loveday E (2014). The incidence and morphological features of pyramidal lobe on thyroid ultrasound. Ultrasound.

[7] Ostrowski P, Bonczar M, Iwanaga J (2023). The prevalence and anatomy of the pyramidal lobe of the thyroid gland: a meta-analysis with implications for thyroid surgery. Clin Anat.

[8] Chaudhary P, Singh Z, Khullar M, Arora K (2013). Levator glandulae thyroideae, a fibromusculoglandular band with absence of pyramidal lobe and its innervation: a case report. J Clin Diagn Res.

[9] Moore K, Persaud TVN (2013). The developing human: clinically oriented embryology 9th edition. 9th edition.

[10] De Felice M, Di Lauro R (2004). Thyroid development and its disorders: genetics and molecular mechanisms. Endocr Rev.

[11] Vayisoglu Y, Ozcan C, Gen R, Eti CM, Sut H, Gorur K (2013). Thyroid isthmus agenesis associated with thyroid papillary carcinoma. J Craniofac Surg.

[12] Loukas M, Merbs W, Tubbs RS, Curry B, Jordan R (2008). Levator glandulae thyroideae muscle with three slips. Anat Sci Int.

[13] Sencar ME, Calapkulu M, Sakiz D (2021). Residual pyramidal lobe increases stimulated thyroglobulin and decreases endogenous thyroid stimulating hormone stimulation in differentiated thyroid cancer patients. Endocr Pract.

[14] Ali AM, Ahmed MA, Saadeldein MS (2024). Anatomical study of thyroid pyramidal lobe among thyroidectomy patients in east Gezira, Sudan. World J Biol Pharm Health.

